# Necdin is a breast cancer metastasis suppressor that regulates the transcription of *c-Myc*

**DOI:** 10.18632/oncotarget.5230

**Published:** 2015-08-19

**Authors:** Minnkyong Lee, Sarah M. Beggs, Derek Gildea, Sujata Bupp, Jens Lichtenberg, Niraj S. Trivedi, NISC Comparative Sequencing Program, Ying Hu, David M. Bodine, Nigel P.S. Crawford

**Affiliations:** ^1^ Genetics and Molecular Biology Branch, National Human Genome Research Institute, NIH, Bethesda MD, USA; ^2^ Computational and Statistical Genomics Branch, National Human Genome Research Institute, NIH, Bethesda MD, USA; ^3^ NIH Intramural Sequencing Center, National Human Genome Research Institute, NIH, Bethesda MD, USA; ^4^ Center for Biomedical Informatics and Information Technology, National Cancer Institute, NIH, Rockville MD, USA

**Keywords:** Necdin, c-Myc, germline polymorphisms, metastasis suppressors, breast cancer

## Abstract

Metastasis is the primary cause of death in breast cancer. Earlier studies using a mammary tumorigenesis mouse model identified *Necdin* (*Ndn*) as a germline modifier of metastasis. Differential expression of *Ndn* induces a gene-expression signature that predicts prognosis in human breast cancer. Additionally, a non-synonymous germline single nucleotide polymorphism (T50C; V17A) in *Ndn* distinguishes mouse strains with differing metastatic capacities. To better understand how hereditary factors influence metastasis in breast cancer, we characterized NDN-mediated transcription. Haplotype analysis in a well-characterized breast cancer cohort revealed that *NDN* germline variation is associated with both *NDN* expression levels and patient outcome. To examine the role of NDN in mammary tumor metastasis and transcriptional regulation, mouse mammary tumor cell lines stably over-expressing either the wildtype 50T or variant 50C *Ndn* allele were generated. Cells over-expressing *Ndn* 50T, but not *Ndn* 50C, exhibited significant decrease in cell invasiveness and pulmonary metastases compared to control cells. Transcriptome analyses identified a 71-gene expression signature that distinguishes cells over-expressing the two *Ndn* allelic variants. Furthermore, ChIP assays revealed c-*Myc*, a target gene of NDN, to be differentially regulated by the allelic variants. These data demonstrate that NDN and the T50C allele regulate gene expression and metastasis efficiency.

## INTRODUCTION

It is estimated in 2015 in the U.S. alone that 231,000 new cases of breast cancer will be diagnosed, and over 40,000 patients will die from this disease [1]. The majority of deaths caused by breast cancer are a result of metastasis [2] with bone and brain being the primary sites of tumor dissemination [3–5]. Molecular characterization of breast cancer has identified predictive gene-expression signatures in breast tumor tissues, greatly advancing our understanding of this disease [6]. Through similar expression signatures in mouse mammary tumors, *Necdin* (*Ndn*) was identified as a driver of extracellular matrix (ECM) gene expression [7], which are a class of genes that are common components of predictive expression signatures [8, 9]. Specifically, *Ndn* was implicated as a driver of ECM gene expression through expression quantitative trait locus (eQTL) mapping in mammary tumors derived from the transgene-positive F1 progeny of the FVB/N-Tg(MMTV-PyVT)634Mul/J (PyMT) mouse model of mammary tumorigenesis and the AKXD recombinant inbred strains of mice. Subsequent *in vitro* and *in vivo* studies demonstrated that modulation of the expression level of *Ndn* impacted the metastatic capacity of a highly aggressive mouse mammary tumor cell line. Most importantly, these earlier studies demonstrated that *Ndn* is a metastasis suppressor that exerts its influence at the germline level.

The gene encoding human NDN is located on chromosome 15q11, a region that is maternally imprinted in Prader-Willi syndrome, a neurogenetic disorder characterized by developmental delays and behavioral abnormalities [10]. Mice with mutated *Ndn* display neonatal lethality and behavioral phenotypes similar to those observed in Prader-Willi syndrome [11, 12]. NDN is a member of the melanoma antigen (MAGE) family which is comprised of over 60 genes that share the highly conserved MAGE homology domain (MHD) [13, 14]. Human NDN is 321 amino acids long and mouse NDN is 325 amino acids long and the two proteins overall share 82% identity. Between human and mouse NDN, the amino-terminal region is less conserved (60% identity) and higher similarity (90% identity) is observed in the functional region of NDN (amino acids 83-292) which contains the MHD [15].

Earlier studies have reported that the expression of NDN is decreased in many different cancer types in comparison to normal tissue, including breast cancer, suggesting that it acts as a tumor suppressor [16]. A gene-expression signature induced by the over-expression of *Ndn* was shown to predict survival in breast cancer patients [7]. While several studies implicate NDN as a possible tumor suppressor, the mechanism behind its role in metastasis is unclear. We hypothesize that NDN, a known transcription factor [17], induces metastasis-predictive gene expression by interacting with chromatin to regulate transcription, and that *Ndn* variants differ in their capacity to regulate the transcription of target genes. In this study using a publically available breast cancer dataset, we demonstrate that *NDN* harbors polymorphisms associated with patient survival. Through *in vitro* and *in vivo* methods using mouse mammary tumor cell lines stably over-expressing variants of *Ndn*, we show that a specific *Ndn* variant acts as a germline metastasis suppressor. Chromatin immunoprecipitation (ChIP) assays demonstrate that NDN achieves this effect most likely by regulating the expression of target genes, such as c-*Myc*, at the transcriptional level. Our work demonstrates that several polymorphisms in *NDN* are significantly associated with clinical outcomes in breast cancer patients, and that these polymorphisms influence the function of NDN as a transcriptional regulator and metastasis suppressor.

## RESULTS

### Germline variation in *NDN* in a well-defined human breast cancer cohort is associated with patient outcome

**Figure 1:**
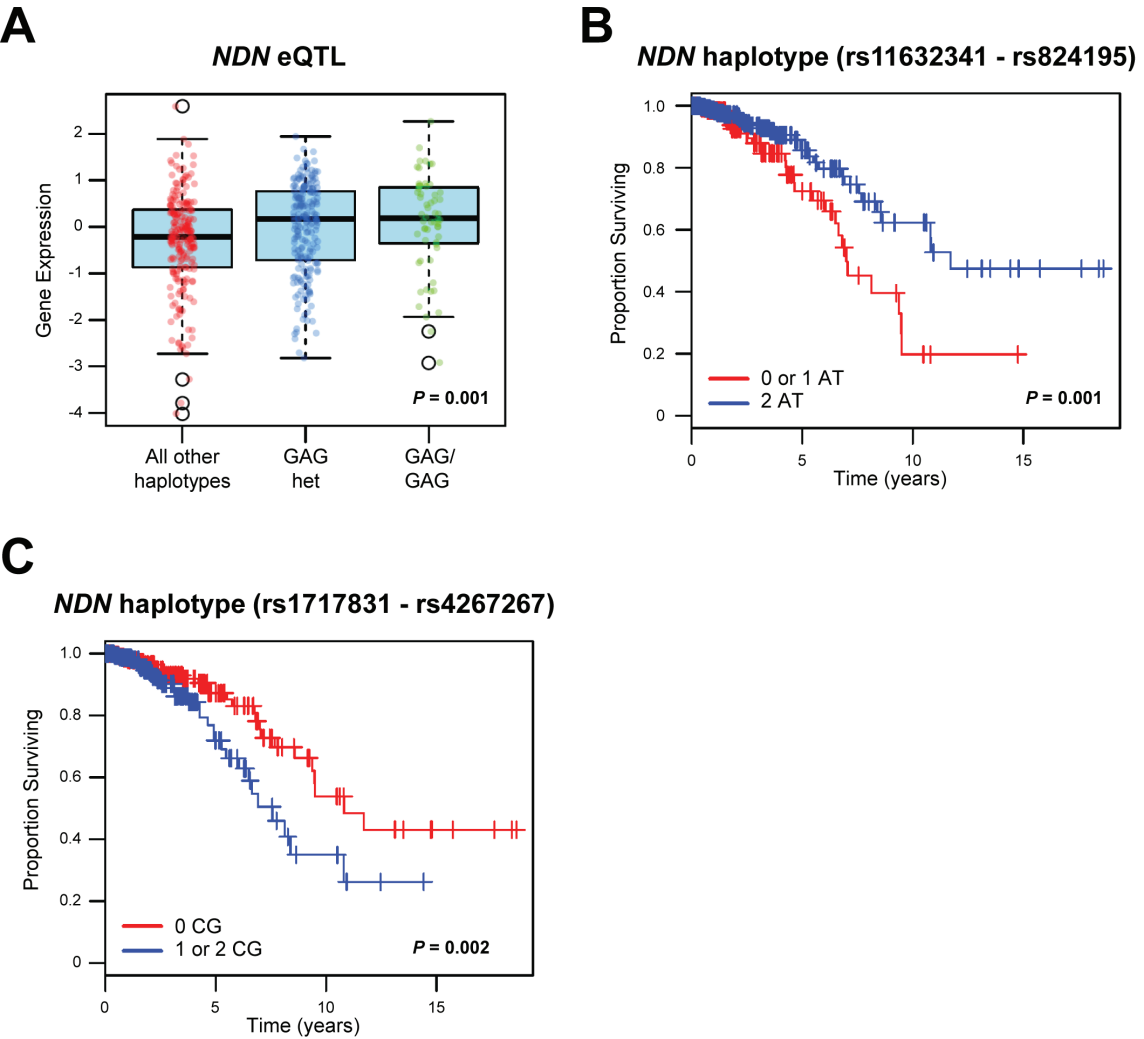
Haplotypes in LD with *NDN* are correlated with *NDN* expression and survival in breast cancer patients **A.**, haplotype analysis of 466 breast cancer patients from TCGA repository demonstrated that a three-marker haplotype (rs1722793 - rs1524843 - rs1781208 GAG) is significantly associated with the expression level of *NDN*. **B.**, **C.**, two marker haplotypes of NDN B, rs11632341 - rs824195 and C, rs1717831 - rs4267267 are significantly associated with survival (B, *P* = 0.001; C, *P* = 0.002) in this breast cancer cohort.

Since *Ndn* was implicated as a germline metastasis susceptibility gene through an eQTL mapping approach in tumors derived from a mouse model of mammary tumorigenesis, we hypothesized that: a) the expression of human *NDN* in breast cancer is influenced by similar patterns of germline variation; and b) common variants in the human *NDN* gene are associated with markers of disease aggressiveness and clinical outcome in breast cancer. To investigate these hypotheses, the frequencies of haplotypes in linkage disequilibrium (LD) with *NDN* (Supplementary Figure 1) were analyzed in 466 breast cancer patients derived from the Cancer Genome Atlas (TCGA) repository. For eQTL analysis, the frequencies of germline haplotypes in LD with *NDN* were correlated with *NDN* gene level RNA-seq expression data in all primary tumors. One haplotype of three single nucleotide polymorphisms (SNPs) (rs1722793 - rs1524843 - rs1781208 GAG), which is located approximately 17 kb downstream of *NDN* and has a frequency of 0.347 in the study population, was significantly associated with the tumor expression level of *NDN* (*P* = 0.001; FDR = 0.048; Figure 1A). Full results for eQTL analysis are shown in Supplementary Table S1.

In addition to demonstrating that the expression level of *NDN* is associated with germline variation, our analyses illustrate that haplotypes in LD with *NDN* are associated with disease aggressiveness and patient survival. Specifically, one haplotype 35 kb upstream and in LD with *NDN* (rs850815 - rs850814 AA) was associated with a more aggressive breast cancer phenotype (Table 1): first, when tumors were classified on their PAM50 status [18], the AA haplotype was more frequent in poor prognosis basal-like tumors (frequency = 0.815 *vs*. 0.447 for all other subtypes; *P* = 1.99×10^−6^; FDR = 1.11×10^−4^); and second, the AA haplotype was more frequent in estrogen receptor (ER) negative compared to ER positive tumors (frequency = 0.716 *vs*. 0.459 for ER+; *P* = 3.57×10^−4^; FDR = 0.020). Finally, two haplotypes in LD with *NDN* were associated with overall survival in this cohort: first, a two-marker haplotype 57 kb downstream of *NDN* (rs11632341 - rs824195 AT) was associated with a significantly better overall survival (*P* = 0.001; Figure 1B); and second, a two-marker haplotype 95 kb downstream of *NDN* (rs1717831 - rs4267267 CG) was found to be significantly associated with a poorer overall survival in breast cancer patients (*P* = 0.002; Figure 1C). Complete results for the association of *NDN* haplotypes with tumor PAM50 basal and ER phenotypes are shown in Supplementary Tables S2 and S3, respectively.

**Table 1:** Association of NDN haplotypes with disease-specific traits

Trait	Haplotype Distance from Gene (bp)	SNPs Defining Haplotype	Haplotype	Frequency		t value	*P*-value	FDR
PAM50	35,587	rs850815 rs850814	AA	Basal	0.815	4.816	1.99×10^−6^	1.11×10^−4^
				Other	0.447			
Tumor ER Status	35,587	rs850815 rs850814	AA	ER-	0.716	−3.597	3.57×10^−4^	0.020
				ER+	0.459			

### A non-synonymous germline variant in mouse *Ndn* modulates *in vitro* colony formation and *in vivo* metastasis

**Figure 2:**
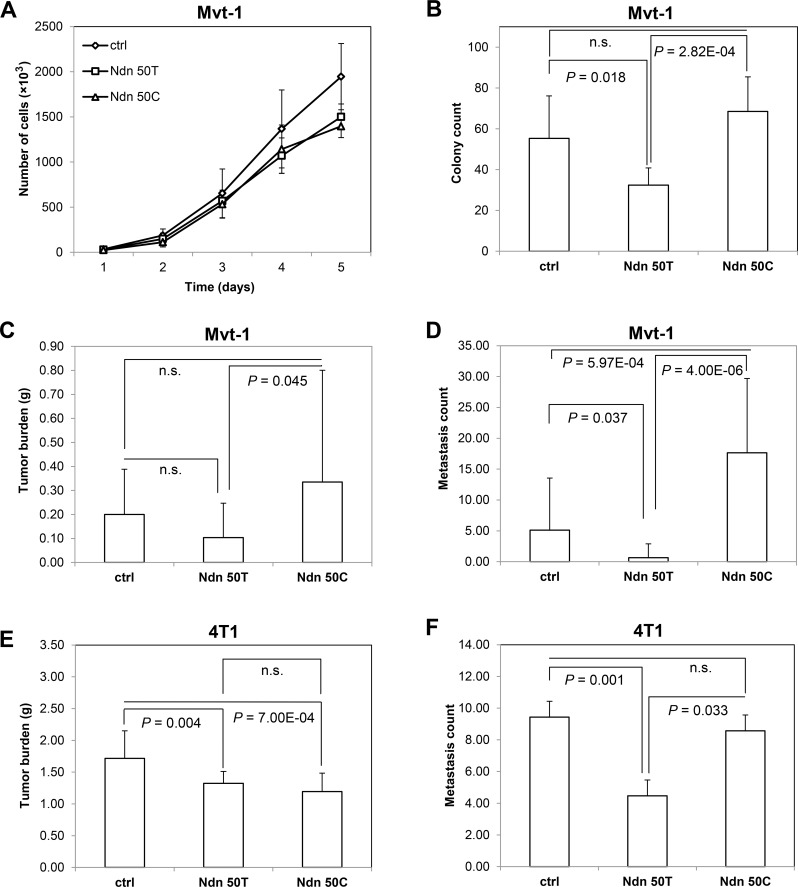
*Ndn* 50T and *Ndn* 50C Mvt-1 cell lines display differential growth rates in soft agar *in vitro* and metastases *in vivo* **A**, cell proliferation assay. B, soft agar assay. Graphs represent the average of two independent experiments and data are represented as A, mean ± SD, and **B**, mean + SD. **C**, tumor burden and **D**, surface pulmonary metastases were quantified after 4 weeks of orthotopic implantation of Mvt-1 cells over-expressing *Ndn* 50T or *Ndn* 50C and control into the mammary fat pad of NU/J (*n* = 20). **E**, tumor burden and F, surface pulmonary metastases after 4 weeks of orthotopic implantation of 4T1 cells over-expressing *Ndn* 50T or *Ndn* 50C and control into the mammary fat pad of BALB/cJ (*n* = 30) mice. Data are represented as mean + SD.

Having demonstrated that germline polymorphisms in human *NDN* are associated with patient outcome, we aimed to characterize the molecular mechanisms linking hereditary variation in this gene with metastasis. As discussed above, mouse *Ndn* was identified as a germline metastasis susceptibility gene using the AKXD recombinant inbred panel, which is derived from the metastasis-prone AKR/J strain and the metastasis-resistant DBA/2J strain. The DBA/2J strain contains a non-synonymous germline SNP (T50C; V17A; rs261911330) that distinguishes it from AKR/J mice. To examine the role of this SNP, we used lentiviral transduction to establish cells stably over-expressing either the wildtype *Ndn* 50T allele or the variant *Ndn* 50C allele in the highly metastatic Mvt-1 mouse mammary tumor cell line. Cells expressing *Ndn* at endogenous levels were used as controls. The *in vitro* growth properties of cell lines over-expressing *Ndn* variants were compared to cells stably transduced with a control lentiviral vector. Analysis of *in vitro* growth rates demonstrated a suppressed growth rate of cells over-expressing either the *Ndn* 50T allele (analysis of covariance [ANCOVA] *P* = 1.30×10^−6^) or the *Ndn* 50C allele (*P* = 3.44×10^−5^) compared to the control (Figure 2A). There was no significant difference in the growth rates in cells over-expressing the *Ndn* 50T allele and the *Ndn* 50C allele. However, soft agar assays performed using Mvt-1 cells stably expressing *Ndn* variants to examine anchorage-independent growth demonstrated a significant decrease in colony growth with the cells over-expressing the wildtype *Ndn* 50T allele compared to both the control (*P* = 0.018) and DBA/2J *Ndn* 50C allele (*P* = 2.82×10^−4^; Figure 2B; Supplementary Figure S2).

To investigate the *in vivo* effects of *Ndn* over-expression, these Mvt-1 cells were implanted into the mammary fat pad of 6-week old female NU/J mice, and tumor growth and pulmonary surface metastasis were quantified at 4 weeks. There was no significant difference in primary tumor burden between either of the two *Ndn* variants and the control. However, there was a significant increase in primary tumor burden in mice implanted with cells over-expressing the wildtype *Ndn* 50T allele (av. tumor burden = 0.10 g ± 0.14 g) compared to those implanted with cells over-expressing the DBA/2J *Ndn* 50C allele (av. tumor burden = 0.34 g ± 0.47 g; *P* = 0.045; Figure 2C). We observed a significant decrease in pulmonary surface metastasis with the over-expression of the wildtype *Ndn* 50T allele (av. pulmonary surface metastasis count = 0.7 ± 2.2; *P* = 0.037), and an averaged 3.5 fold increase with the over-expression of DBA/2J *Ndn* 50C (av. pulmonary surface metastasis count = 17.7 ± 12.0; *P* = 5.97×10^−4^) compared to that of the control (av. pulmonary surface metastasis count = 5.1 ± 8.4; Figure 2D). To confirm these results we stably over-expressed either allelic variant or a control vector in the highly metastatic 4T1 mouse mammary tumor cell line, and implanted these into the mammary fat pad of syngenic 6-week old female BALB/cJ mice. Quantification of tumor growth and pulmonary metastases was performed after 4 weeks. In 4T1 cells, there was a decrease in tumor burden with the over-expression of both the wildtype *Ndn* 50T allele (av. tumor burden = 1.32 g ± 0.19 g; *P* = 0.004) and the DBA/2J *Ndn* 50C allele (av. tumor burden = 1.19 g ± 0.29 g; *P* = 7.00×10^−4^) variants compared to control (av. tumor burden = 1.72 g ± 0.43 g; Figure 2E). As was the case with the Mvt-1 cell line, over-expression of wildtype *Ndn* 50T in 4T1 caused a significant decrease in pulmonary surface metastasis compared to control mice (av. pulmonary surface metastasis count = 4.5 ± 3.7 *vs*. 9.4 ± 3.9 control; *P* = 0.001; Figure 2F). However, with 4T1 cells there was no significant difference in pulmonary surface metastasis between controls and cells over-expressing DBA/2J *Ndn* 50C (av. pulmonary surface metastasis count = 8.6 ± 6.0; Figure 2F). Thus, with both cell lines, we observed a significant decrease in pulmonary metastases with the over-expression of wildtype *Ndn* 50T but not DBA/2J *Ndn* 50C compared to control (Figure 2D, 2F).

### Ndn allelic variants differentially regulate the expression of *c-Myc*

**Figure 3:**
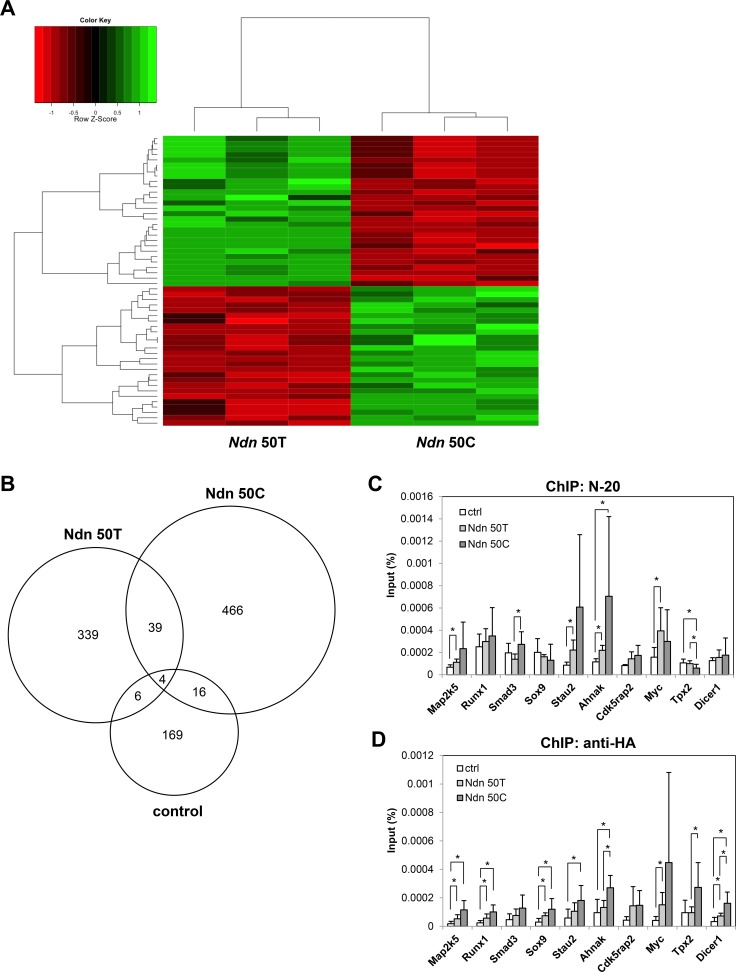
*Ndn* T50C allelic variant differentially regulates gene expression **A.**, microarray analysis of gene expression in Mvt-1 cells stably over-expressing either *Ndn* 50T or *Ndn* 50C. **B.**, Venn diagram representing unique and common binding regions for NDN identified by ChIP-seq in 4T1 cells over-expressing *Ndn* 50T or *Ndn* 50C and control. **C.**, **D.**, verification of NDN binding regions in 4T1 cells through ChIP-qPCR in 4T1 cells over-expressing *Ndn* 50T or *Ndn* 50C and control using **C.**, N-20 or **D.**, anti-HA antibodies. Graphs represent the average of two independent experiments each performed in triplicate and data are represented as mean + SD. *; *P* ≤ 0.05.

Previous studies have demonstrated that NDN is a multifunctional protein that can directly bind to DNA to function as a transcription factor [17] or indirectly regulate transcription by interacting with other well-known transcription factors such as E2F1 and p53 to modulate their transcriptional activities [19, 20]. Microarray analysis of Mvt-1 cells stably over-expressing wildtype *Ndn* 50T or DBA/2J *Ndn* 50C showed that the allelic variant generated a 71-gene expression signature (Figure 3A). Gene enrichment analysis using AmiGO [21] showed that one of the top biological processes in which these genes function is regulation of dendritic cell differentiation, suggesting a role in immune responses. Full gene enrichment analysis results are shown in Supplementary Table S4.

To identify specific target genes of NDN and examine the role of the *Ndn* T50C SNP in transcriptional regulation, we performed genome-wide ChIP-sequencing (ChIP-seq) in 4T1 cells stably over-expressing either HA-tagged wildtype *Ndn* 50T or DBA/2J *Ndn* 50C. NDN was immunoprecipitated using an antibody targeting endogenous NDN (N-20), and NDN binding peaks were called using MACS [22]. These analyses identified a total of 1039 binding peaks, of which, 339 peaks were unique to the *Ndn* 50T allele, 466 peaks unique to the *Ndn* 50C allele, and 169 peaks unique to the control (Figure 3B). A complete list of peaks identified in ChIP-seq analyses for the *Ndn* 50T, *Ndn* 50C, and control 4T1 cell lines are shown in Supplementary Tables S5, S6, and S7, respectively. For validation of ChIP-seq data, over 50 sets of primers were designed for peak regions. Of those tested through quantitative real-time PCR (qPCR), ten primer sets for ten different genes were selected for ChIP-qPCR using the NDN N-20 antibody based on the quality of amplification and dissociation curves. Overall, our ChIP-qPCR data confirm the findings of our ChIP-seq analysis (Figure 3C). To validate the specificity of the anti-NDN N-20 antibody, a second antibody recognizing the HA tag was used to immunoprecipitate ectopically expressed NDN and ChIP-qPCR analysis was performed for the same ten genes. A significant degree of overlap between the anti-NDN N-20 and anti-HA antibodies was observed in these ChIP-qPCR analyses (Figure 3D).

**Figure 4:**
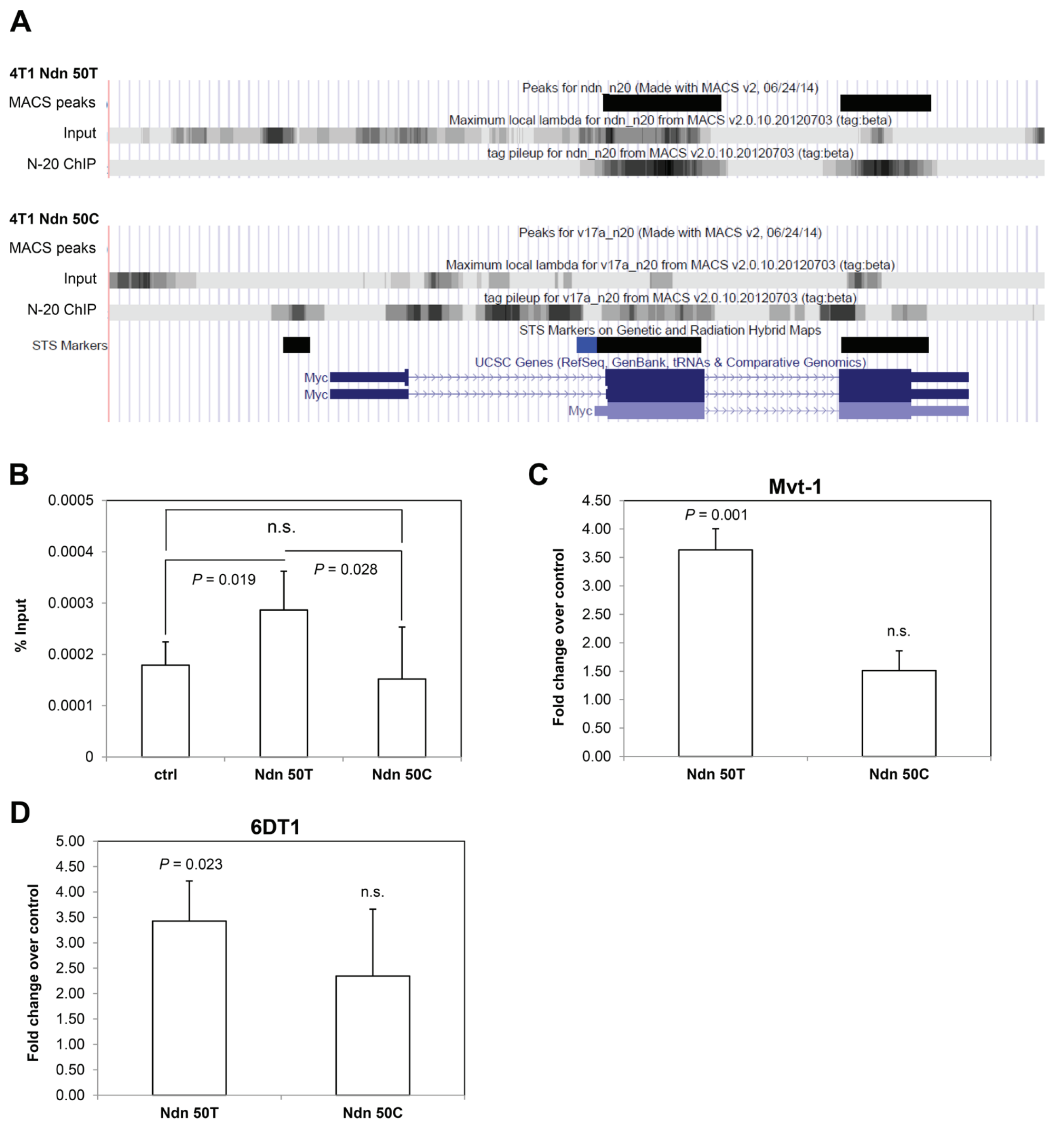
Ectopic expression of *Ndn* 50T, but not *Ndn* 50C, increases *c-Myc* expression in mouse mammary tumor cell lines **A.**, NDN binding peaks detected in c-*Myc* coding regions in ChIP-seq in 4T1 cells over-expressing *Ndn* 50T but not *Ndn* 50C. **B.**, NDN binds to c-*Myc* in ChIP-qPCR in Mvt-1 cells over-expressing *Ndn* 50T but not *Ndn* 50C. **C.**, **D.**, expression of c-*Myc* detected by qPCR in three stable clonal isolates over-expressing either *Ndn* 50T or *Ndn* 50C compared to control in **C.**, Mvt-1 cells and **D.**, 6DT1 cells. Graphs represent the average of two independent experiments and data are represented as mean + SD. *P*-values shown in comparison to control.

We were particularly interested to note that our ChIP-seq data suggested that the wildtype *Ndn* 50T but not the DBA/2J *Ndn* 50C bound the coding region of the proto-oncogene c-*Myc* (Figure 4A), which is also known to be a metastasis suppressor [23]. Having used ChIP-qPCR to validate this differential binding of allelic variants to c-*Myc* in the 4T1 cell line (Figure 3C, 3D), we validated these findings in Mvt-1 cells stably over-expressing either *Ndn* allelic variant. As was the case in 4T1 cells, *Ndn* 50T, but not the *Ndn* 50C variant, was found to bind to c-*Myc* (Figure 4B). We analyzed the expression of c-*Myc* in various cell lines ectopically expressing either allelic variant to explore the effect of this differential binding of *Ndn* allelic variants. On qPCR analysis, over-expression of *Ndn* 50T was associated with a three-fold increase in c-*Myc* expression levels compared to control in both Mvt-1 and 6DT1 mammary tumor cell lines. However, over-expression of *Ndn* 50C did not cause a significant change in the expression level of c-*Myc* (Figure 4C, 4D).

Finally, having demonstrated that mouse *Ndn* allelic variants differentially regulate the expression of c-*Myc*, we examined whether germline variation in human *NDN*, was associated with c-*MYC* expression. Specifically, we characterized associations between haplotypes in LD with human *NDN* and the expression level of c­-*MYC* in TCGA breast cancer cohort. One haplotype of two SNPs (rs7170719 - rs17117524) in LD with *NDN* was found to be associated with the level of *c-MYC* within primary tumors (Supplementary Figure S3).

## DISCUSSION

*Ndn* was identified as a germline metastasis suppressor in an earlier study that sought to identify hereditary factors driving the expression of breast cancer metastasis-associated transcripts [7]. The eQTL mapping element of this previous study, which involved mapping metastasis-associated transcripts in genetically diverse PyMT mice, only indicated that *Ndn* was a modifier of metastasis and not the directionality of the effect. Thus, in this earlier study, the wildtype *Ndn* 50T was ectopically expressed in a highly aggressive mammary tumor cell line and demonstrated that *Ndn* suppressed metastasis. Further, it was demonstrated that a gene-expression signature induced by ectopic expression of *Ndn* in this cell line was associated with patient outcome in multiple breast cancer cohorts. To clarify the role of *Ndn* in metastasis, the aims of the current study are threefold: first, and most critically, to confirm that *Ndn* is a germline modifier of patient outcome in human breast cancer; second, to explore how germline variation in mouse *Ndn* modifies its functionality in order to more clearly understand its role in metastasis susceptibility in this model system; and third, to functionally characterize *Ndn* in order to define a mechanism by which it impacts metastasis. We believe that we have achieved these aims in the current work, and overall this study allows for a much clearer understanding of how *Ndn* acts to influence metastasis at the germline level. We have demonstrated in the small, yet well characterized TCGA human breast cancer cohort that SNPs in putative regulatory regions are not only associated with the expression of *NDN* in primary breast tumors, but are also associated with markers of patient outcome and survival. We have also demonstrated that germline variation in mouse *Ndn* is of importance with regards to metastasis, with a non-synonymous variant present in mouse strains with highly divergent metastatic capacities being strongly associated with differential tumor dissemination in both *in vitro* and *in vivo* analyses. Finally, we propose that the mechanism through which *Ndn* is impacting metastasis is, at least in part, through regulation of the metastasis suppressor c-*Myc*.

There are, however, a number of issues that warrant further explanation, the first of which is the paradoxical metastasis enhancing effect of the *Ndn* 50C allele, which is derived from the DBA/2J strain. This effect is paradoxical since it is well established that DBA/2J is a low metastatic capacity strain, at least when crossed with the PyMT mouse model of mammary tumorigenesis [24]. However, as noted above, the eQTL mapping approach used to identify *Ndn* as a metastasis modulator generally cannot be used to infer the directionality of an effect, only involvement in the phenotype of interest. The overall influence of germline variation upon metastasis in a given mouse strain (or individual human) is likely the sum of the effects of metastasis enhancing alleles minus the effects of metastasis suppressive alleles. Thus, the effect of the metastasis suppressive alleles carried by DBA/2J will outweigh the effects of metastasis enhancing alleles. Additionally, it should be noted that the identification of metastasis enhancing alleles in a metastasis suppressive strain is not unprecedented, with earlier studies demonstrating the existence of such alleles in DBA/2J [25].

The second aspect of this study that requires clarification is the equivalency of variants seen in mouse *Ndn* and human *NDN*. To be more specific, although our studies implicate the T50C coding polymorphism within mouse *Ndn* as being associated with metastasis, it appears that there are no coding polymorphisms in human *NDN* with a minor allele frequency of > 1% in any population. The most likely explanation for this observation is that the coding SNP in mouse *Ndn* phenocopies the regulatory variants seen in human *NDN*. Indeed, when one considers the wildtype mouse *Ndn* 50T allele in isolation, it is clear that higher levels of this have a metastasis suppressive effect (Figure 2D, 2F). Thus, variation of the expression level of mouse *Ndn* alone is sufficient to impact mammary tumor metastasis, at least in those strains lacking the variant 50C allele. In humans, the situation is somewhat similar, in that our eQTL analysis of TCGA breast cancer cohort demonstrates that the expression level of *NDN* in primary tumors is associated with germline haplotypes in LD with *NDN*. However, closer scrutiny of this argument reveals another limitation of this study: although we identified *NDN* haplotypes as being associated with the expression levels of this gene, the same haplotypes were not associated with markers of clinical outcome in the TCGA cohort. However, when one examines the results from our association and eQTL analyses, it is apparent that haplotypes significantly associated with aggressive disease clinical variables approach statistical significance in eQTL analyses, and vice versa. For example, when one considers the rs850815 - rs850814 AA haplotype associated with PAM50 basal tumors (Table 1; referred to as haplotype ID ‘X15_466.42.AA’ in Supplementary Table S2), the uncorrected *P*-value is statically significant for eQTL analysis for the same haplotype (*P* = 0.027; FDR = 0.379). This likely indicates that the current association study is underpowered, and we acknowledge the need to replicate these associations in additional breast cancer cohorts where genotype and gene expression data are available. Additionally, it is clear that haplotypes associated with different traits are all in LD with one another (Supplementary Figure 3).

In this study, we have demonstrated that over-expression of the mouse wildtype *Ndn* 50T allele, but not the DBA/2J 50C allele, induces increased expression of c-*Myc*. The outcome with the wildtype *Ndn* 50T allele is in accordance with previous studies demonstrating that NDN is a transcriptional regulator of c-*Myc* [17, 26]. c-*Myc* is a well-characterized proto-oncogene that promotes tumorigenesis by increasing cellular proliferation. Paradoxically, however, a prominent study by Liu and colleagues [23] demonstrated that c-*MYC* is in fact a metastasis suppressor, with over-expression of c-*MYC* suppressing both the *in vitro* invasiveness and *in vivo* metastatic capacity of human breast cancer cell lines. Surprisingly, over-expression of either *Ndn* allelic variant suppressed the growth rate of the Mvt-1 cell line (Figure 2A), which suggests that the increased expression of c-*Myc* observed with over-expression of *Ndn* is associated with the metastasis-suppressing properties,rather than the pro-tumorigenic properties of *c-Myc*. Additionally, it is plausible that *Ndn* influences other pathways that suppress cell growth rate, and act to counterbalance the pro-proliferative effect of c-*Myc*. Thus, in keeping with earlier studies [23] the decrease in metastasis observed with over-expression of *Ndn* 50T is similar to that observed with the over-expression of c-*Myc* [23]. We do, however, acknowledge that further experimentation will be required to fully understand the relationship between *Ndn* and c-*Myc*. For example, knockdown of c-*Myc* in conjunction with expression of the wildtype *Ndn* 50T could demonstrate that the metastasis suppressive effect of wildtype *Ndn* are solely dependent upon c-*Myc*. However, in our hands, knockdown of c-*Myc* in the Mvt-1 and 6DT1 cell lines has resulted in substantial cell death, which is presumably due to the fact that both cell lines originate from mammary tumor explant-cell cultures derived from the MMTV-c-*Myc* transgenic mouse model of mammary tumorigenesis [27]. Therefore, a more quantitative approach to c-*Myc* knockdown utilizing new technology will be required to address this issue, which we argue are beyond the scope of the current work. Thus, while we acknowledge that it is difficult to conclude that the increased expression of c-*Myc* is solely responsible for the inhibition of metastasis observed in cells over-expressing *Ndn*, it is striking that the DBA/2J *Ndn* 50C allele that lacks the ability to suppress metastasis also appears to lack the ability to transcriptionally regulate c-*Myc*.

In conclusion, this study has demonstrated that *Ndn* is a metastasis suppressor that acts at the germline level, and that it modulates metastasis primarily through transcriptional regulation of c-*Myc*. Overall, this adds to the growing body of evidence that illustrates the importance of host background on outcome in a wide variety of tumors including breast cancer [25, 28, 29], prostate cancer [30, 31], and melanoma [32]. In the era of precision medicine, it may be the case that consideration of host-specific hereditary factors will add an additional dimension of personalization of therapy, beyond that which is possible by studying the landscape of somatic mutations within a primary tumor alone. Future efforts will concentrate on identifying additional hereditary factors, which, like *Ndn*, play a prominent role in modulating outcome in breast cancer.

## MATERIALS AND METHODS

### SNP haplotype analysis

Data from 466 breast cancer patients in TCGA/BCRA cohort was used for SNP haplotype analysis. SNP Hardy-Weinberg equilibrium (HWE) *P*-values were estimated with PLINK. Of the 77 SNPs within 100 kb of *NDN*, 15 SNPs were excluded from the analysis due to being uninformative or if the HWE *P-*value < 10^−4^, and 62 were analyzed. For haplotype analysis, LD blocks were constructed using Haploview, and fastPHASE was performed to estimate haplotypes for each individual based on the LD blocks on NIH biowulf super cluster computer system (http://biowulf.nih.gov). Survival analysis was performed using the Cox model of the survival package in R [33, 34]. A generalized linear model (glm) was used to perform the association analyses between haplotypes and either *NDN* gene expression or clinical subtypes. Age and PC1, PC2, and PC3 were included as covariates in the glm. Correction for multiple testing was performed using the Benjamini-Hochberg FDR [35] by the MULTITEST package of R. Clinical subtypes analyzed included ER and PR status, PAM50 subtypes, P53 mutant status, PIK3CA mutation status, MAP3K1 mutation status, and MAP2K4 mutation status.

### Generation of stable cell lines

Cells stably over-expressing either *Ndn* 50T or *Ndn* 50C were generated in mouse mammary tumor cell lines Mvt-1, 4T1, and 6DT1 using lentiviral transduction. For control cell lines, an empty pLenti vector was used. Cells were selected using 10 μg/mL puromycin for Mvt-1 and 6DT1, or 5 μg/mL puromycin for 4T1 cells. Clonal isolates were derived through serial dilution, and expression of *Ndn* 50T or *Ndn* 50C was confirmed by qPCR (Supplementary Figure S4).

### Orthotopic mammary fat pad implantation

Six to 8-week-old female NU/J and BALB/cJ mice were purchased from Jackson Laboratory (Bar Harbor, ME, USA). NU/J mice were injected with 10^6^ cells of either control, *Ndn* 50T, or *Ndn* 50C Mvt-1 cells (*N* = 20 per group) and BALB/cJ mice were injected with 10^6^ cells of either control, *Ndn* 50T, or *Ndn* 50C 4T1 cells (*N* = 30 per group) as previously described [36]. Mice were euthanized four weeks after injections, and pulmonary surface metastases were counted and tumors were weighed. All animal experiments were performed in compliance with the National Human Genome Research Institute Animal Care and Use Committee's guidelines.

### Growth curve and soft agar assay

For growth curve analysis, 2.5×10^4^ cells for each clonal isolate were plated in 12 well plates. Cells from each clonal isolate were counted in duplicate on a daily basis for 5 days. The growth rates of each cell line were analyzed by comparing scaling parameters (i.e., slopes) between two groups (control *vs*. *Ndn* 50T, control *vs. Ndn* 50C, and *Ndn* 50T *vs. Ndn* 50C). In this comparison, ANCOVA was used to compare the two general linear models by testing the effect of a group on a cell count while controlling for the effect of the time (days). For soft agar assays, four clonal isolates of each of the control, *Ndn* 50T, or *Ndn* 50C Mvt-1 cells were plated in 0.33% agar in 12-well plates at 4×10^3^ cells per well. Each isolate was plated in duplicate and allowed to grow in 37°C, 5% CO_2_. Cells were then stained with 0.005% crystal violet for colony counting.

### RNA isolation and qRT-PCR

Total RNA was prepared from cells using RNeasy Plus Mini Kit (QIAGEN) following manufacturer's protocol. RNA concentration was measured using NanoDrop (Wilmington, DE, USA). Reverse transcription was performed using iScript DNA Synthesis Kit (Bio-Rad, Hercules, CA, USA) and qRT-PCR reactions were performed using ABI Fast SYBR Green Master Mix (Life Technologies, Grand Island, NY, USA) as previously described [36].

### Microarray and data analysis

Total RNA from Mvt-1 clonal isolates stably over-expressing *Ndn* 50T or *Ndn* 50C was extracted using RNeasy Plus Mini Kit (QIAGEN), then processed using Affymetrix Mouse Gene 1.0 ST Array (Santa Clara, CA, USA) according to the manufacturer's protocol. Data obtained were analyzed using Partek Genomics Suite (St. Louis, MO, USA) and heatmaps were generated using R [37]. Microarray data are publicly available through Gene Expression Omnibus (GEO accession no. GSE65824).

### Chromatin immunoprecipitation (ChIP)

ChIP assays were performed as previously described [38]. Briefly, after pre-clearing with Protein G Sepharose beads (GE Healthcare, Piscataway, NJ, USA), antibodies targeting endogenous NDN (N-20, Santa Cruz, Santa Cruz, CA, USA), HA (anti-HA, Roche) or IgG (12-370, Millipore, Billerica, MA, USA) was added to the sonicated cell lysates and incubated at 4°C for 2 hours. Protein G Sepharose beads were then added and incubated overnight. The following day, immunocomplexes were washed twice with lysis buffer, once with high salt buffer (50 mM HEPES-KOH pH 7.5, 500 mM NaCl, 1 mM EDTA, 0.1% Triton X-100, 0.1% sodium deoxycholate), twice with LiCl buffer (10 mM Tris-HCl pH 8.0, 0.25 M LiCl, 0.5% NP-40, 0.5% sodium deoxycholate, 1 mM EDTA) and once with TE buffer, followed by elution in TE buffer containing 1% SDS. Samples were incubated at 65°C overnight for reverse-crosslinking, and treated with RNAse A and proteinase K the next day. ChIP DNA was purified using QIAquick PCR Purification Kit (QIAGEN) according to manufacturer's protocol. The DNA obtained was analyzed either by high-throughput sequencing or qPCR. All ChIP-qPCR reactions were performed in duplicate using ABI Fast SYBR Green Master Mix (Life Technologies) mentioned above.

### High-throughput sequencing analysis

ChIP-seq was performed on the Illumina HiSeq2500 platform as previously described [38]. Sequence reads from the ChIP-enriched samples and corresponding input were 51 bps in length. ELAND was used to map sequences to the mouse genome (mm9), and alignments were outputted in BAM format. The software bamToBed (BedTools suite v. 2.10.1; http://code.google.com/p/bedtools), was used to convert the BAM-formatted mapping data to BED format. Reads that mapped to satellite DNA were removed using the script bed_intersect.py (bx-python 0.5.0; “-v” option). A BED file containing genomic locations of satellite DNA was obtained from mm9 RepeatMasker data available at UCSC. These filtered BED files were used as data input for the Model-based Analysis of ChIP-seq version 2 (MACS2 v. 2.0.10.20120703) software. When calculating ChIP peaks, mapping data from the ChIP input sequences were supplied to MACS2 as a control. The following parameters were used when executing MACS2 with the “macs callpeak” command: effective genome size = 1.87×10^9^; band width = 300; model fold = [5,50]; q value cutoff = 0.05; range for calculating regional lambda: 1000 bps and 10000 bps.

## SUPPLEMENTARY MATERIAL FIGURES AND TABLES
















