# Supplementary material for: Necdin is a breast cancer metastasis suppressor that regulates the transcription of c-Myc
Source: Oncotarget. 2015 Aug 19;6(31):31557–68. doi: 10.18632/oncotarget.5230 (PMC4741624; doi:10.18632/oncotarget.5230)
Supplement: Supplementary file 1 [file oncotarget-06-31557-s001.pdf]

Necdin is a breast cancer metastasis suppressor that regulates the transcription of *c-Myc*

Supplementary Material

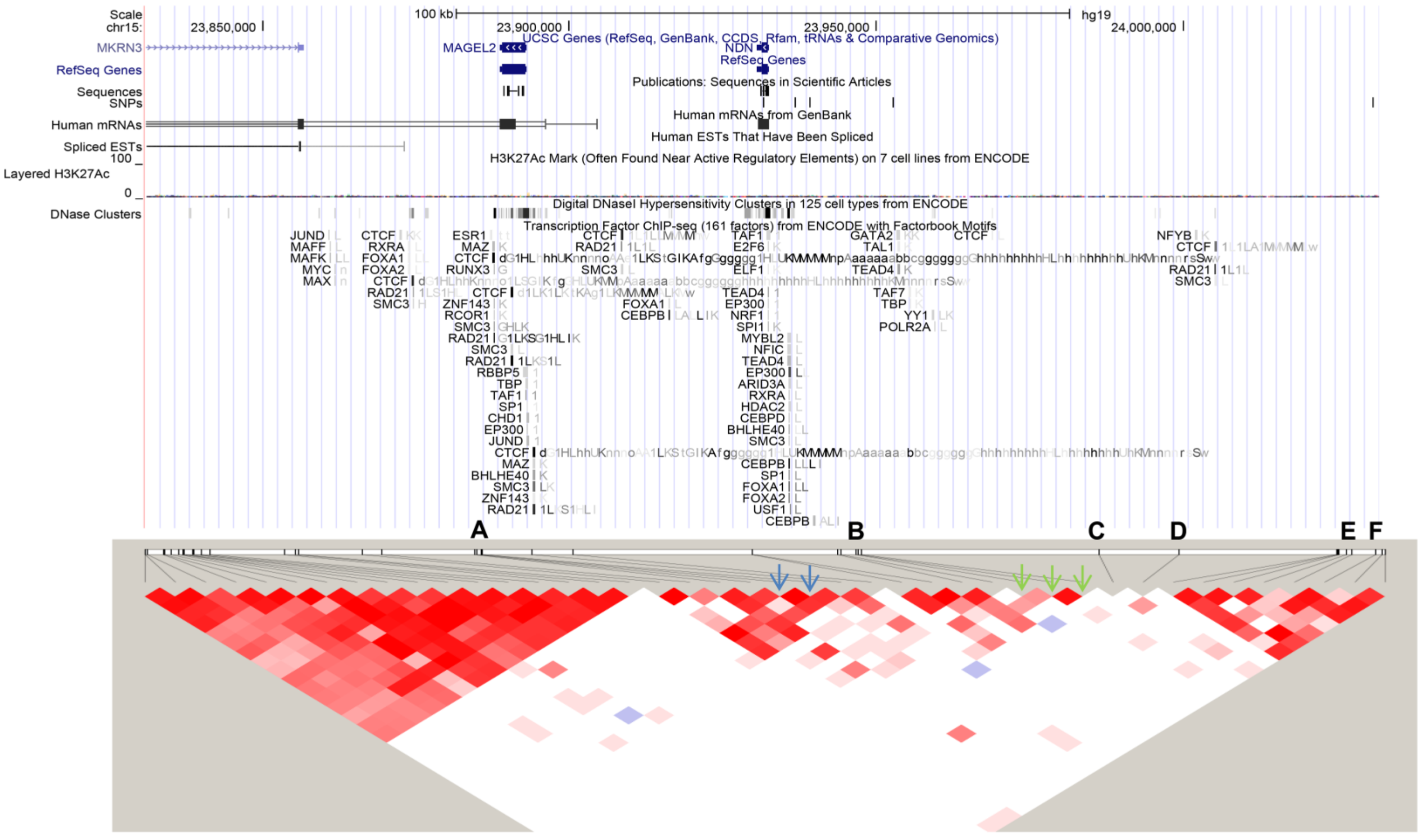

A; rs850815, rs850814, **B**; rs1722793, rs1524843, rs17812081, **C**; rs11632341, **D**; rs824195, **E**; rs1717831, **F**; rs4267267

Supplementary Figure S1. Representation of haplotypes in LD with *NDN*.

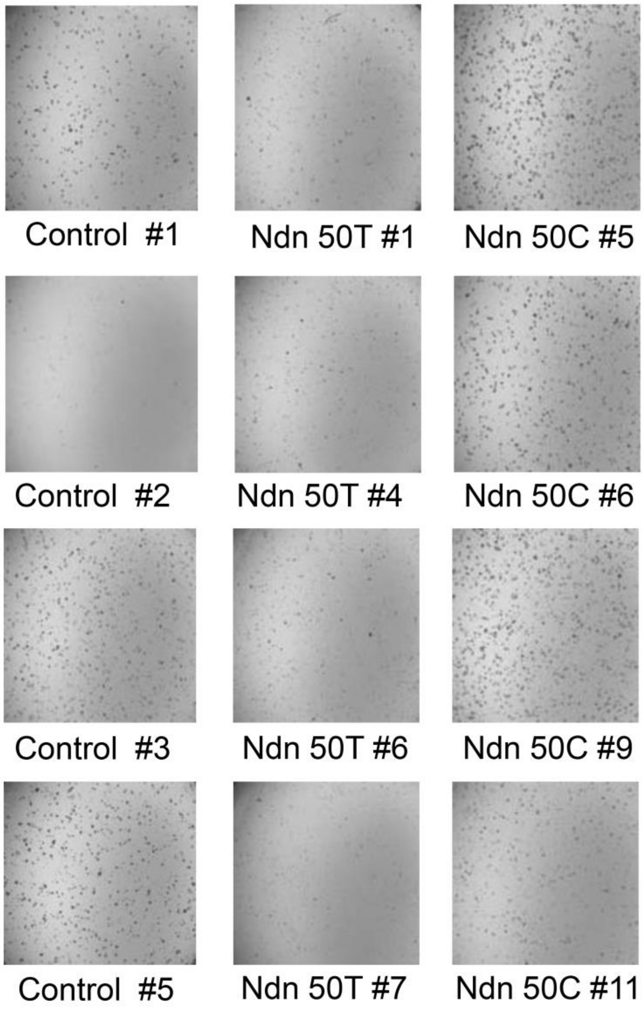

Supplementary Figure S2. Representative images captured at 7 days after culture with 0.005% crystal violet staining (numbers indicate individual clones for each cell line).

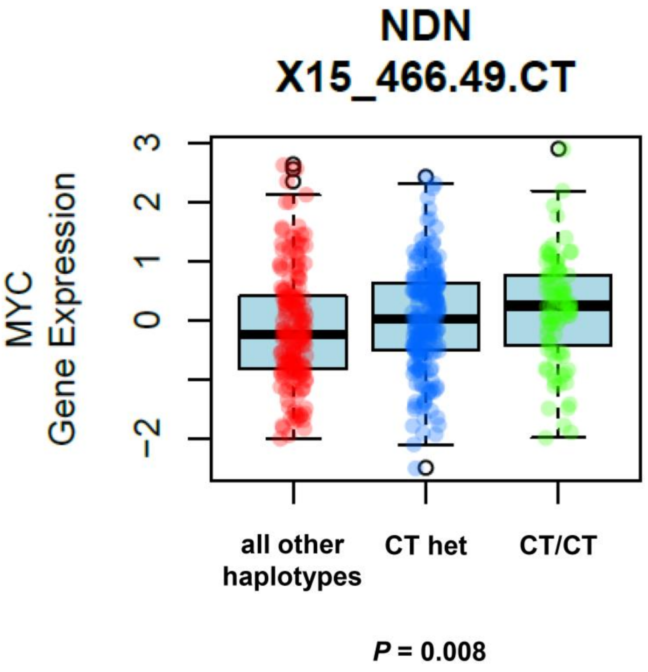

Supplementary Figure S3. A two-marker haplotype (rs7170719 - rs17117524) in LD with *NDN* is associated with the expression of *c-MYC* in TCGA cohort.

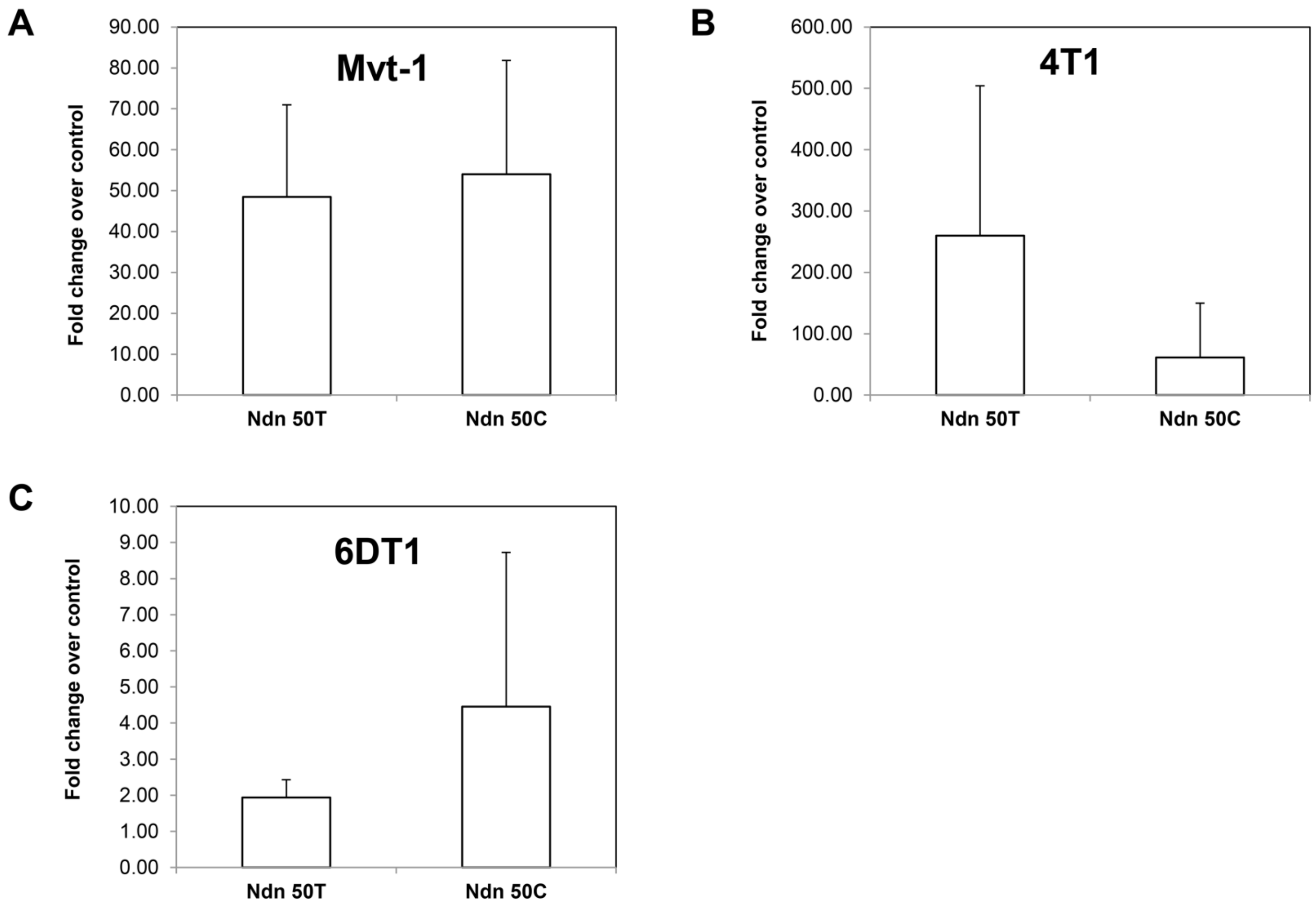

Supplementary Figure S4. Over-expression of Ndn allelic variants in clonal isolates was confirmed by qPCR. A, Mvt-1 cells. B, 4T1 cells. C, 6DT1 cells. Graphs represent the average of three clonal isolates and data are represented as mean + SD.
